# Maca against Echinococcosis?—A Reverse Approach from Patient to In Vitro Testing

**DOI:** 10.3390/pathogens10101335

**Published:** 2021-10-15

**Authors:** Tanja Karpstein, Sheena Chaudhry, Solange Bresson-Hadni, Michael Hayoz, Ghalia Boubaker, Andrew Hemphill, Reto Rufener, Marc Kaethner, Isabelle Schindler, Yolanda Aebi, Antonio Sa Cunha, Carlo R. Largiadèr, Britta Lundström-Stadelmann

**Affiliations:** 1 Department of Infectious Diseases and Pathobiology (DIP), Institute of Parasitology, Vetsuisse Faculty, University of Bern, 3012 Bern, Switzerland; tanja.karpstein@swisstph.ch (T.K.); sheena.chaudhry@vetsuisse.unibe.ch (S.C.); ghalia.boubaker@vetsuisse.unibe.ch (G.B.); andrew.hemphill@vetsuisse.unibe.ch (A.H.); reto.rufener@vetsuisse.unibe.ch (R.R.); marc.kaethner@vetsuisse.unibe.ch (M.K.); 2 French National Reference Center for Echinococcosis, Parasitology-Mycology Laboratory, Besançon University Hospital, University Bourgogne Franche-Comté, 25000 Besançon, France; dr.bresson.hadni@wanadoo.fr; 3 Gastroenterology and Hepatology Division and Division of Tropical and Humanitarian Medicine, Geneva Faculty of Medicine, Geneva University Hospital, 1205 Geneva, Switzerland; 4 Department of Clinical Chemistry & Center for Laboratory Medicine, Bern University Hospital (Inselspital), 3010 Bern, Switzerland; michael.hayoz@insel.ch (M.H.); isabellePaula.schindler@insel.ch (I.S.); yolanda.aebi@insel.ch (Y.A.); carlo.largiader@insel.ch (C.R.L.); 5 Centre Hépato-Biliaire, Assistance Publique Hôpitaux de Paris Hôpital Paul Brousse, 94800 Villejuif, France; antonio.sacunha@aphp.fr

**Keywords:** *Lepidium meyenii*, *Echinococcus multilocularis*, Maca, alveolar echinococcosis, treatment, phytotherapy, medicinal plant, UHPLC-MS/MS, metabolite levels

## Abstract

Drug-based treatment of alveolar echinococcosis (AE) with benzimidazoles is in most cases non-curative, thus has to be taken lifelong. Here, we report on a 56-year-old male AE patient who received standard benzimidazole treatment and biliary plastic stents, and additionally self-medicated himself with the Peruvian plant extract Maca (*Lepidium meyenii*). After 42 months, viable parasite tissue had disappeared. Based on this striking observation, the anti-echinococcal activity of Maca was investigated in vitro and in mice experimentally infected with *Echinococcus multilocularis* metacestodes. Albendazole (ABZ)-treated mice and mice treated with an ABZ+Maca combination exhibited a significantly reduced parasite burden compared to untreated or Maca-treated mice. As shown by a newly established UHPLC-MS/MS-based measurement of ABZ-metabolites, the presence of Maca during the treatment did not alter ABZ plasma levels. In vitro assays corroborated these findings, as exposure to Maca had no notable effect on *E. multilocularis* metacestodes, and in cultures of germinal layer cells, possibly unspecific, cytotoxic effects of Maca were observed. However, in the combined treatments, Maca inhibited the activity of ABZ in vitro. While Maca had no direct anti-parasitic activity, it induced in vitro proliferation of murine spleen cells, suggesting that immunomodulatory properties could have contributed to the curative effect seen in the patient.

## 1. Introduction

The cestode *Echinococcus multilocularis* (small fox tapeworm) causes alveolar echinococcosis (AE), the highest ranked foodborne parasitic disease in Europe [1]. *E. multilocularis* is endemic in the Northern hemisphere, affecting at least 18,500 new human cases of AE each year and inflicting more than 666,400 disability-adjusted life years (DALYs) [2]. AE poses an uncontrolled health problem especially in developing and resource-poor regions [3], and is regarded as a neglected and emerging disease [4,5,6].

In its natural life cycle, *E. multilocularis* is transmitted between definitive hosts (including canids such as foxes, dogs, and raccoon-dogs) and intermediate hosts such as voles [7]. However, humans, captive monkeys, dogs, and other mammals can also be infected accidentally by ingesting *E. multilocularis* eggs released by final hosts. The parasite oncosphere is then released from the egg in the intestine and migrates through the intestinal wall, reaches the bloodstream, and finally ends up in the affected organ, which is most often the liver. Here, development to the metacestode takes place. Metacestodes are characterized by an unlimited proliferative potential, thus they represent the disease-causing stage. The highly infiltrative growth of metacestodes can cause severe organ dysfunction. Non-specific symptoms are induced in the progressive stage of AE, and they include abdominal pain, jaundice, cholestasis, hepatomegaly, fever, anemia, weight loss, and pleural pain [3,8]. At the advanced stage of AE, and if treatment fails, the disease will lead to the death of the patient.

Curative treatment of AE can be reached by radical surgical resection of the parasite, but this can only be applied if the medical infrastructure is given (thus not in resource-poor settings) and it cannot be performed when AE is diagnosed at a late stage of infection [9,10]. Due to the risk of recurrence, surgery is combined with temporal medical treatment and long-term monitoring [3]. If complete resection is not possible, AE patients receive drug treatment based on the benzimidazoles (BMZ) mebendazole (MBZ) or albendazole (ABZ). Whereas treatment with BMZ has drastically improved the survival rates of AE patients, treatment failures due to adverse side effects, including life-threatening hepatotoxicity, are frequently observed [9,11]. Further, BMZ treatment has to be taken life-long, as these drugs are only parasitostatic, and in most cases do not kill the parasite [3]. Given these shortcomings of BMZs, novel alternative drug treatment options are urgently needed.

The development of reliable techniques for the in vitro culture of *E. multilocularis* metacestodes and respective stem cells has paved the way for the development of a standardized drug-screening platform, which allows for objective quantification of drug-induced effects on the parasite [12]. AE mouse models allow to further assess compounds of interest in vivo [12]. To date, most studies have focused on the repurposing of anti-cancer compounds, anti-infective drugs, and also the potential application of immunotherapeutics against AE in mice. An additional group of growing interest for repurposing are natural products and medicinal plant extracts. Medicinal plants are low in costs and show strong pharmacological potential, exhibit anti-oxidant, anti-inflammatory, or anti-proliferative potential, and are therefore increasingly tested against various morbidities [13]. Medicinal plants have been used for millennia, but only over the last few decades did molecular approaches of drug discovery allow for identification of active fractions and components, and toxicological as well as pharmacodynamical studies have been performed [14,15].

**Figure 1:**
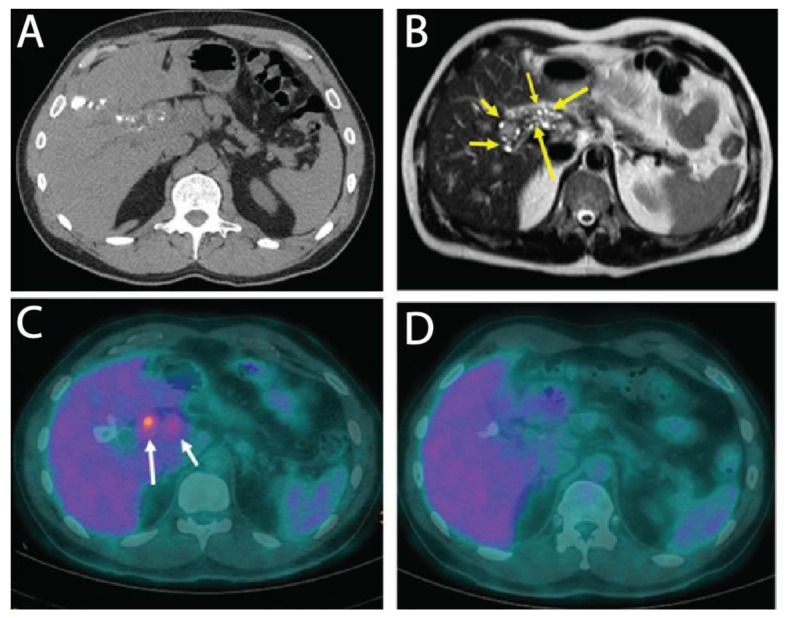
Radiological images of human AE liver. (**A**) CT image of human AE patient in July 2013, at time of diagnosis showing an infiltrative lesion with scattered calcifications in the right lobe of the liver and extending to the hilum; (**B**) T2 weighted MR image showing several hyper-T2 microcysts in the perihilar area (arrows) testifying to an active AE lesion; (**C**) FDG-PET at time of diagnosis, early acquisition image (1 h after FDG injection): obvious FDG uptake around the perihilar parasitic mass (arrows); (**D**) FDG-PET in 2018, under ABZ therapy for 54 months, late acquisition image (3 h after FDG injection): no perilesional activity.

**Figure 2:**
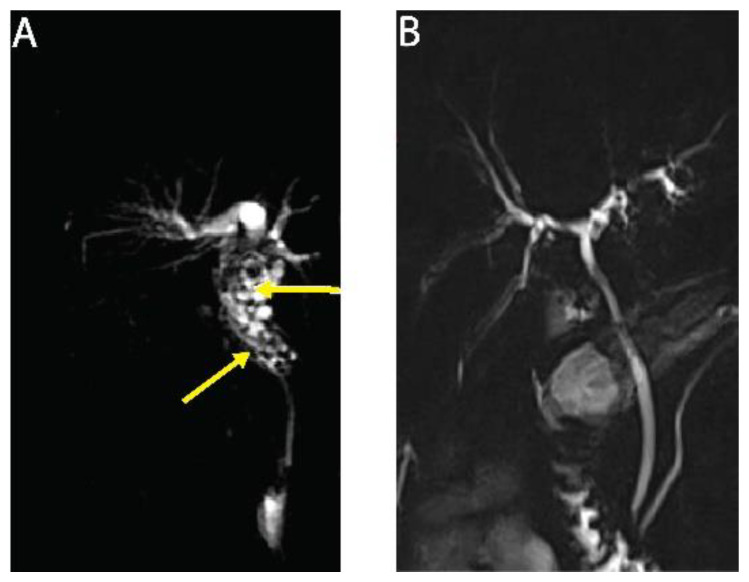
Cholangio-MRI images of human AE patient. (**A**) in July 2013, at time of diagnosis of a severe liver AE revealed by jaundice due to choledochus involvement. Numerous HyperT2 microcysts surrounding the upper part of choledochus (arrows). Above, note the enlargement of the intrahepatic right bile duct due to the impaired bile flow. (**B**) in February 2017 under ABZ therapy for 42 months. Complete disappearance of the hyper-T2 microcysts. In parallel, very favorable clinical and biological evolution, anti-Em18 antibodies negativation and perilesional activity disappearance on PET-CT evaluation (see Figure 1).

Here, we report on a clinical case of AE, where the involvement of a medicinal plant extract has been implicated. In mid-July 2013, a 56-year-old man working as executive in industry and living near Paris without any history of significant health issues presented an isolated jaundice associated with fatigue. Abdominal ultrasonography and computed tomography (CT) indicated an aberrant liver mass located in the hilar region associated with intra-hepatic bile ducts dilatation (Figure 1A). A cholangiocarcinoma was suspected and the patient was referred to the hepato-biliary reference center at Paul Brousse Hospital (France). At admission a liver and cholangio-magnetic resonance imaging (-MRI) was performed. It confirmed the presence of a perihilar infiltrative tissular mass. However, hyper-T2 sequences showed multiple microcystic structures within the lesion (Figure 1B and Figure 2A). This aspect, associated with many other lesions in the right liver (segment V and VIII) with mixed composition (cystic and calcified areas) when rereading the CT images, raised suspicion of AE. Specific serology confirmed this diagnosis (with 26–28 kDa and 16 and 18 kDa positivity on Western Blot for confirmation. Thus, continuous ABZ therapy was introduced (400 mg bid). The patient had travelled a lot for professional reasons, mainly in Asia and South America but not in areas that are endemic for AE. However, before moving to Paris, he was living in Eastern France from 2001–2007, in a village located in Lorraine, an area known to be endemic for AE [16]. There, he had an open vegetable garden, which was frequently accessed by foxes that also came very close to the patient’s house. The patient did not own dogs or cats. A first PET-CT in August 2013 showed strong hypermetabolic perilesional foci surrounding the hilar lesion (Figure 1C); another hypermetabolic focus was described along the choledochus until the cephalic pancreas. There was no perilesional activity in the right liver but this first PET-CT did not include the late acquisitions images (+ 3 h) that are recommended for AE lesions evaluation [17]. Moreover, a more extended lesion check-up was performed: a thoracic CT-scan and cerebral MRI did not reveal AE metastases. The PNM stage (8) was P4N0M0, stage IIIb.

Due to the hilar localization of the parasitic mass with extension along the biliary tree, a liver transplantation associated with ABZ appeared as the sole solution to cure this young patient [8]. However, upon the advice from the French Reference Center for AE in Besançon University Hospital, a medical option was recommended. An endoscopic biliary plastic stent was placed at the end of July 2013 while continuing ABZ therapy. Pharmacological monitoring helped to maintain plasma ABZ-sulfoxide (ABZ-SOX) levels (+4 h) between 1 and 3 µmol/L as previously recommended [18,19]. Ursodeoxycholic acid was added (500 mg bid) in order to reduce the risk of stent obstruction. Cholangitis occurred at 10 days after stenting, which was treated by antibiotics and stent replacement.

Subsequently, the situation evolved favorably without any new clinical event. In September 2013 jaundice disappeared. Biological follow-up indicated a stable normalization of liver enzymes during the 6-year follow-up. ABZ was very well tolerated with excellent observance and optimal pharmacological results (concentrations always maintained between 2 and 4 µmol/L with no posology adaptation due to good tolerance). In April 2014, the biliary stent was replaced by three juxtaposed plastic stents allowing calibration of the main stenosis located on the left biliary duct. Regular stent replacements were performed every 3 to 6 months until May 2015, then stents were definitively removed. The clinical, biological course remained uneventful, and the patient returned to completely normal activities. Furthermore, the specific serological follow-up for AE was extremely favorable, as it showed a continuous decrease in Em2+ ELISA index (from 1.27 initially to negativity at 0.49 and 0.44 in September 2018 and 2019, respectively) and anti-Em18 reactivity also disappeared. Very interestingly and totally unexpectedly, so close to the diagnosis and initiation of ABZ, MRI follow-up indicated a complete disappearance of hyper-T2 microcysts at 42 months post ABZ initiation (Figure 2B). PET-CT sequential evaluation with 1 and 3 h acquisition following a specific protocol for AE [17] indicated a regular decrease in perilesional activity to complete negativation during 2015–2018 (Figure 1D). This was confirmed in 2019. The patient had a strong demand to discontinue ABZ treatment. After a multidisciplinary meeting at the Besançon Reference Center, the decision for ABZ interruption after an 80 month duration was validated in March 2020 under strict follow-up. The first evaluation at 6 months post-treatment interruption, in September 2020, indicated negative specific serological results, normal liver values, and the patient’s physical condition remained excellent. The situation remained unchanged in September 2021, with AE serology negative, normal liver enzymes, and total bilirubinemia.

This rapid favorable course of a very severe AE under ABZ, which usually only acts as a parasitostatic, was surprising. In fact, serology and imaging results indicated that metacestode death occurred as soon as three years post ABZ initiation. By questioning the patient, clinicians were informed that he had self-medicated himself since 2013 with the plant extract Maca (*Lepidium meyenii*). The extract was purchased from Naturoconcept (Brunoy, France), and he ingested five pills of 500 mg per day, corresponding to brown roots from a Peruvian source. The patient decided to take this compound on self-medication at time of AE diagnosis, as an immunostimulant. He stopped the intake of Maca for 3 months at the time of ABZ interruption in March 2020. He has taken Maca again thereafter on a sequential scheme (3 months interruption followed by 3 months uptake).

Based on this isolated, but interesting case, studies were carried out on the efficacy of Maca and combined ABZ–Maca therapy in mice, and the potential direct effect of Maca exposure in *E. multilocularis* metacestodes and germinal layer cells in vitro. In particular potential differences in mouse serum levels of ABZ metabolites in the presence/absence of Maca were studied using a newly established UHPLC-MS/MS-based technique that allows analyses of minute amounts of serum. In addition, the direct effects of Maca exposure on the capacity of splenocyte proliferation were carried out to investigate potential immunomodulatory activity of the medicinal plant extract.

## 2. Materials and Methods

If not stated otherwise, all reagents and chemicals were purchased from Sigma-Aldrich (Buchs, Switzerland). Maca (Bio) was from Naturoconcept (Brunoy, France). As stated by the company, the roots of *Lepdium meyenii* were harvested, cut in pieces, dried, and were processed to powder without any further extraction.

For cell culture, DMEM without phenol red, FCS and Trypsin-EDTA were purchased from Bioswisstec (Schaffhausen, Switzerland). DMEM, penicillin and streptomycin were from Thermo Fisher Scientific (Zug, Switzerland).

For UHPLC—MS/MS, ABZ sulfoxide (ABZ-SOX) 95%, ABZ sulfone (ABZ-SON) 95% as well as the internal standards [^2^H_7_]-ABZ-SOX 95% and [^13^C,^2^H_3_]-ABZ-SON 98% were obtained in solid form from Alsachim (Illkirch Graffenstaden, France). Acetonitrile, methanol, ammonium acetate, and formic acid 99% (all UPLC–mass spectrometry grade) were from Biosolve (Valkenswaard, the Netherlands). Ultrapure water was produced in-house using a Milli-Q station from Merck Millipore (Darmstadt, Germany). An analyte-free human serum (DC Mass Spect Gold serum MSG4000) was purchased from Golden West Biologicals (Temecula, CA, USA). 

### 2.1. Mice and Ethics Statement

Animals were purchased from Charles River Laboratories (Sulzheim, Germany) and used for experimentation after 2 weeks of acclimatization. The animals weighed 23.5–24.5 g in average. BALB/c mice were maintained in a 12 h light/dark cycle, controlled temperature of 21–23 °C, and a relative humidity of 45–55%. Food and water were provided ad libitum. All animals were treated in compliance with the Swiss Federal Protection of Animals Act (TSchV, SR455), and the procedures used in this study were approved by the Animal Welfare Committee of the canton of Bern under the license number BE126/17.

### 2.2. Efficacy Assessment of Maca against Secondary AE in Mice

Thirty-six 8-week-old BALB/c mice were applied for efficacy assessment of Maca against murine AE. In vitro cultured *E. multilocularis* metacestodes were used for infection, as described earlier [20]. Each mouse received 100 µL parasite suspension in PBS (50%) by i.p. injection (secondary infection AE model). After infection, mice were randomly allocated into 4 groups of 9 animals each (4 and 5 animals per cage) and were marked by ear tattooing for individual identification. Five weeks after infection, treatment was initiated. The 4 treatment groups were as follows: (i) ABZ, 200 mg/kg, p.o. gavage in corn oil, 5 days per week (*n* = 9); (ii) Maca, 33 mg/kg, p.o. gavage in corn oil, 5 days per week (*n* = 9); (iii) ABZ+Maca, 200 and 33 mg/kg respectively, combined p.o. gavage in corn oil, 5 days per week (*n* = 9); (iv) corn oil (vehicle) control, p.o. gavage, 5 days per week (*n* = 9). All animals were treated for 6 weeks. The mouse weight was assessed every second week, well-being of the animals was checked daily, and blood samples were taken at 1, 3, and 6 weeks of treatment (see below). At the end of the treatment period, all mice were euthanized by CO_2_. The parasite tissue was carefully resected from the peritoneal cavity and weighed. Statistical analysis of the data was performed in R Studio version 1.4.1103. Data distribution was checked by Shapiro–Wilk test. Kruskal–Wallis chi-squared test was performed to analyze if there were any differences between the treatment groups, and for pairwise comparisons Wilcoxon rank sum test was applied. *p* < 0.01 was considered as highly significant, *p* < 0.05 as significant.

### 2.3. Assessment of ABZ-Metabolite Serum Levels in Mice Treated against Secondary AE

To assess the levels of the ABZ-metabolites ABZ-SOX and ABZ-SON in treated AE-mice, the serum levels were assessed by a newly developed ultrahigh-performance liquid chromatography tandem mass spectrometry (UHPLC-MS/MS) approach. The steps of (i) sampling, (ii) preparation of solutions, (iii) sample preparation, (iv) UHPLC-MS/MS, and data analysis are described below.

#### 2.3.1. Sampling of Mouse Sera

In the in vivo experiment described above, blood samples were taken from the tail vein during treatment (weeks 1 and 3), and heart-blood was taken at the endpoint (week 6) of treatment, for subsequent analysis of ABZ-SOX and ABZ-SON concentrations. At each of these time points, blood samples were retrieved 1, 4, and 8 h after ABZ dosage from 3 mice in each group. The blood samples were allowed to coagulate for 30 min at room temperature, then they were centrifuged at 3000× *g* for 10 min, 4 °C, to retrieve serum samples, which were stored at −20 °C until further analysis. 

#### 2.3.2. Preparation of Calibrator, Quality Control, and Internal Standard Solutions for UHPLC-MS

Separate solutions of the following drugs and the isotope-labeled analogue were prepared: ABZ-SOX and the internal standard [^2^H_7_]-ABZ-SOX were directly dissolved in methanol at a concentration of 1 mg/mL. ABZ-SON and the internal standard [^13^C,^2^H_3_]-ABZ-SON were solubilized at a concentration of 1 mg/mL in dimethyl sulfoxide/methanol (1:4, *v*/*v*). A mixed stock solution of non-deuterated compounds at 6 mg/L for ABZ-SOX and 4 mg/L for ABZ-SON in methanol were used for the preparation of calibrators. In the same way, an independent mixed stock solution was made for the quality controls.

Seven calibrator spiking solutions were prepared by diluting the stock solutions with methanol to final concentrations of 0.09, 0.19, 0.38, 0.75, 1.5, 3.0, and 6.0 mg/L for ABZ-SOX and 0.06, 0.13, 0.25, 0.50, 1.0, 2.0, and 4.0 mg/L for ABZ-SON. The same procedure was repeated for four quality control spiking solutions with the final concentrations of 0.14, 0.56, 1.9, and 4.7 mg/L for ABZ-SOX and 0.09, 0.38, 1.3, and 3.1 mg/L for ABZ-SON in methanol.

Furthermore, a mixed internal standard stock solution containing 1.2 mg/L ABZ-SOX and 0.8 mg/L ABZ-SON was prepared in methanol. The daily used working solution for the precipitation was prepared by diluting the internal standard stock solution 1:20 with acetontrile (*v*/*v*).

#### 2.3.3. Sample Preparation

For protein precipitation and analyte extraction of calibrators and quality controls, 25 µL of the calibrator and quality control spiking solutions, at the appropriate concentration, followed by 180 µL acetonitrile containing the internal standards ([^2^H_7_]-ABZ-SOX, [^13^C,^2^H_3_]-ABZ-SON) were added to 40 µL DC Mass Spect Gold serum (Golden West Biologicals).

Concerning protein precipitation and analyte extraction of patient samples, 25 µL of methanol, followed by 180 µL acetonitrile, containing the internal standards ([^2^H_7_]-ABZ-SOX, [^13^C,^2^H_3_]-ABZ-SON), were added to 40 µL serum. After incubation and mixing for 10 min, the samples were centrifuged at 4000× *g* and 20 °C for 15 min. Next, 20 µL supernatant was diluted to 480 µL with methanol containing 1% formic acid. The prepared samples were sealed and stored in the autosampler at 10 °C until analysis. 

#### 2.3.4. UHPLC-MS/MS for Measurement of ABZ-Metabolite Levels

Next, 1 µL of the extracted samples were injected into a reverse-phase CORTECS C8 Column, 90Å, 1.6 µm, 2.1 mm × 100 mm (Waters Corp. Milford, MA, USA) with a gradient mobile phase comprising 0.1% ammonium acetate with 1% formic acid (A) and acetonitrile containing 0.1% ammonium acetate with 1% formic acid (B). Each sample was resolved for 3.7 min at a flow rate of 0.5 mL/min with the linear gradient 0–1.2 min from 10 to 98% B; 1.2–2.2 min 98% B, and 10% B for 1.5 min. The column temperature was 45 °C. The eluent was introduced by electrospray ionization into the mass spectrometer (Xevo TQ-S, Waters Corp.), operating in positive ion electrospray ionization mode (ESI+). The capillary voltage was set to 3000 V and the Source Offset to 50 V. The dissolving gas flow was set to 1200 L/h and the temperature to 650 °C. The cone gas flow was 200 L/h, and the source temperature was set to 150 °C. 

To establish the appropriate multiple reaction monitoring (MRM) conditions for the individual compounds, the cone voltage was optimized to maximize the intensity of the protonated molecular species [M + H]+ and the collision energy (eV) was adjusted to optimize the signal for the most abundant product ions, which were subsequently used for MRM analysis (Table 1). 

#### 2.3.5. UHPLC-MS/MS Data Analysis

The data processing was performed with TargetLynx, available in the MassLynx software (version 4.1, Waters Corp.) by integration of the area under the specific MRM chromatograms in reference to the integrated area of the isotope-labeled analogue. 

The calibration curves were constructed using concentrations ranging from 0.2–12.8 µmol/L of ABZ-SOX, respectively 0.125–8.0 µmol/L of ABZ-SON by using linear regression with a 1/x weighting factor. Mean values and standard deviations were calculated in Excel.

### 2.4. In Vitro Assessment of Maca against E. multilocularis Metacestodes and Germinal Layer Cells

In order to assess the in vitro effects of Maca and ABZ alone and in combination on cultured metacestode vesicles and germinal layer (GL) cells, the following assays were performed in vitro: (i) phosphoglucose isomerase (PGI) assay; (ii) Alamar Blue cell viability assay; (iii) GL cell viability assay, and (iv) transmission electron microscopy (TEM). The PGI assay (i) measures the damage induced on whole *E. multilocularis* metacestode vesicles, whereas the Alamar Blue assay (ii) shows the impact on cell viability within metacestodes. In the GL cell viability assay (iii) the impact on the viability of extracted GL cells is assessed. Transmission electron microscopy (TEM) (iv) shows structural changes and damage induced by treatment of metacestodes. 

#### 2.4.1. PGI Assay

In vitro coculture of *E. multilocularis* (isolate H95) metacestodes with Reuber rat hepatoma cells and the PGI assay were performed as described previously [21]. In short, in vitro cultured metacestode vesicles of 6 to 10 weeks of age and approximately 4 to 6 mm in size were washed in PBS and taken up in double the volume of DMEM without phenol red, including penicillin (100 U/mL) and streptomycin (100 µg/mL). Parasites were distributed into a 48-well plate to a total of 1 mL per well. ABZ was prepared as 40 mg/mL stock in DMSO, Maca as 100 mg/mL stock in DMSO, and Tx-100 as 20% stock in PBS. These substances were added as follows: (i) Maca (50 µg/mL); (ii) Maca (50 µg/mL) in combination with ABZ (10.6 µg/mL = 40 µM); (iii) ABZ alone (40 µM); (iv) Tx-100 (0.1%) as positive control; (v) DMSO (0.1%) as negative control. Every condition was tested in triplicate. Following culture at 37 °C, 5% CO_2_, during 5 and 12 days, culture supernatants were assessed for PGI-activity [21]. Relative enzyme activities were calculated as percentages of the Tx-100 control and are given as mean values and standard deviations for each triplicate. Three independent assays were carried out. 

For an independent PGI-inhibition analysis, vesicle fluid from *E. multilocularis* metacestodes was incubated with Maca at 50 µg/mL, with ABZ at 10.6 µg/mL (i.e., 40 µM), or with a combination of both, and compared to mock incubation with DMSO only. PGI-activity was measured in triplicates and repeated three times independently. Mean values and standard deviations calculated as described above.

#### 2.4.2. Alamar Blue Metacestode Viability Assay

The Alamar Blue assay was applied as described earlier, on parasites of the above-described setup after 12 days of drug incubation [22]. In short, metacestode vesicles that were cultured in the presence of Maca and/or ABZ, as well as the respective DMSO and Tx-100 controls (as described above under (i)), were broken up/disrupted by passing them through a 1 mL pipette. Resazurin was added to 20 mg/mL to each well, and was mixed with the disrupted metacestodes. The change in fluorescence was measured over 5 h of incubation on the EnSpire multilabel reader (Perkin Elmer). Relative viability was calculated based on the DMSO control, and mean values and standard deviations are given. Three independent assays were carried out. 

#### 2.4.3. GL Cell Viability Assay

GL cell viability assay was assessed as described [22,23]. In short, conditioned medium (cDMEM) was prepared based on DMEM supplemented with 10% FCS, 100 U/mL penicillin, 100 μg/mL streptomycin, and 5 μg/mL tetracycline, and 50 mL medium was placed into a T175 flask containing either 10^6^ Reuber rat hepatoma cells during 6 days, or 10^7^ cells during 4 days, both at 37 °C / 5% CO_2_, under humid atmosphere. The two conditioned media were mixed 1:1 and sterile filtered, and stored at 4 °C until further use. For GL cell isolation, *E. multilocularis* metacestode cultures (isolate H95) of at least one year of age were washed in PBS, and hepatoma cells removed by short incubation in water. Metacestode vesicles were mechanically broken by pipette, and vesicle fluid was washed away. After an additional PBS-washing step, GL cells were removed from the vesicle tissue by incubation in Trypsin-EDTA, and occasional shaking for 30 min. The cell solution was filtered through a 30 µm sieve (Sefar AG, Heiden, Switzerland), and the remaining vesicle tissue was reincubated and sieved again until no more cells detached. Califerous corpuscules were removed by centrifugation at 50× *g*. The GL suspension was centrifuged at 600× *g*, 4 °C, for 10 min. The resulting GL cell pellet was taken up in cDMEM, and diluted to an OD_600_ of 0.1 (equal to 1 AU/µL). Then, 1000 AU were seeded in 5 mL cDMEM and incubated overnight at 37 °C, humid atmosphere, under N_2_ atmosphere. The following day, cells were resuspended, and 2000 AU of cells were unified and incubated for another 3 h at 37 °C under nitrogen atmosphere. Subsequently, cells were distributed at 12.5 AU/well in a square, flat bottom, black 384-well plate in 12.5 µL cDMEM per well. ABZ was added to 10.6 µg/mL, Maca to 50 µg/mL, and respective combinations of Maca and ABZ. DMSO (0.1%) served as negative control, the compound MMV665807 (0.3 µg/mL, i.e., 1 µM) as positive control [22]. Each condition was tested in quadruplicate. Plates were incubated for 3 and 5 days at 37 °C, humid atmosphere, under N_2_ atmosphere. Thereafter, the viability of GL cells was assessed by CellTiter-Glo luminescent assay. Next, 25 µL of CellTiter-Glo (Promega, Dübendorf, Switzerland) including 1% Tx-100 was added and cell aggregates disrupted by pipetting. Measurements were performed on a EnSpire multilabel reader (Perkin Elmer, Waltham, MA, USA). Mean values and standard deviations were calculated and are expressed in relation to the DMSO control. The assay was performed once.

#### 2.4.4. Ultrastructural Examination by TEM

One additional sample of each condition was prepared for TEM as described (Hizem et al., 2019). In short, specimens were prefixed in 2% glutaraldehyde in 0.1 M sodium cacodylate buffer, pH 7.3 for 1 h at room temperature, and post-fixation was performed in 2% osmium tetroxide in 0.1 M sodium cacodylate buffer during 2 h at room temperature. Samples were washed in distilled water and treated with Uranyless (Electron Microscopy Sciences, Hatfield, PA, USA) for 30 min, washed in distilled water, dehydrated by sequential incubations in ethanol (30, 50, 70, 90, and three times 100%), and subsequently embedded in Epon-812 resin. Polymerization of the resin was carried out overnight at 60 °C. Sections (80–90 nm of thickness) were cut on a Reichert and Jung ultramicrotome, loaded onto 300-mesh formvar-carbon-coated nickel grids (Plano GmbH, Marburg, Germany), and were stained with Uranyless and lead citrate. Samples were inspected on a CM12 TEM operating at 80 kV.

### 2.5. In Vitro Assessment of Immunomodulatory Properties of Maca

To assess whether Maca exhibited an immunomodulatory effect on freshly isolated murine splenocytes (a), cells were either left unstimulated (control resting cells), stimulated with Concanavalin A (Con A), stimulated with lipopolysaccharide (LPS), or incubated with Maca at different concentrations. In addition, to check whether Maca could affect proliferation of Con A- or LPS-activated lymphocytes (b), splenocytes were either left unstimulated, induced by Con A or LPS, or Con A or LPS in combination with Maca.

For readout of both tests (a and b), splenocyte viability was indirectly assessed by Alamar Blue assay, and splenocyte proliferation by BrdU assay. Thus, for the in vitro assessment of a potential immunomodulatory effect of Maca, mouse splenocytes had to be isolated (i), stimulating agents were prepared (ii), for readout of viability the Alamar Blue assay (iii) was applied, and for measurement of cell proliferation the BrdU assay (iv) was used.

#### 2.5.1. Isolation of Mouse Splenocytes

Single-cell suspension from mouse spleen was prepared exactly as previously described [24,25]. Briefly, spleens of three 14-week-old female BALB/c mice were aseptically removed, minced, and passed through 40 µm cell strainers. After erythrocytes lysis, remaining cells were counted using a hemacytometer and trypan blue. The spleen cell suspension was adjusted to a final concentration of 2 × 106 cells/mL in a complete RPMI-1640 (Thermo Fisher Scientific) containing 10% FCS, 100 U/mL penicillin, 100 μg/mL streptomycin, 2mM of L-glutamine, and 55 µM of β-mercaptoethanol. Spleen cells were then seeded in 96-well plates at a density of 2 × 10^5^ cells/well in 100 µL medium per well. After stimulation, cells were incubated for 48 h at 37 °C with 5% CO_2_, humid atmosphere.

#### 2.5.2. Preparation of Stimulating Agents

Con A was used as known mitogen leading to polyclonal activation of T- lymphocytes. Con A (stock solution at 0.5 mg/mL) was applied at a final concentration of 5 µg/mL. Bacterial LPS was used as a potent stimulant of B-cells, LPS (stock solution at 1 mg/mL) was applied at a final 10 µg/mL. Cyclosporine A (Cs A, stock solution at 8.31 mM) was used at 1 µM as a powerful immunosuppressive drug. Maca was prepared as a stock of 8 mg/mL in DMSO, and tested at final concentrations of 8, 1, and 0.12 µg/mL.

#### 2.5.3. Alamar Blue Splenocyte Viability Assay

Alamar Blue assay on splenocytes was carried out as described previously [24,25]. Briefly, after 48 h of incubation at 37 °C in the presence of 5% CO_2_, 0.02 mL of a resazurin solution (0.1 mg/mL) was added to each well and fluorescence was measured at 0 and 5 h. Results represent the mean fluorescence of five replicates ± standard deviation. To determine whether observed differences between groups were significant, data were subjected to Student’s t-test analysis. *p*-values below 0.05 were considered as significant, below 0.01 as highly significant.

#### 2.5.4. BrdU ELISA

The 5-bromo-2′-deoxyuridine (BrdU) cell proliferations assay was performed as described previously [24,25]. Shortly, BrdU was added to splenocytes cultures 18 h before the end of the experiment, and incorporation of BrdU was measured using BrdU cell proliferation kit (QIA58, Merck Millipore). Results represent the mean absorbance of three replicates ± standard deviation. To determine whether observed differences between groups were significant, data were subjected to Student’s t-test analysis. *p*-values below 0.05 were considered as significant, below 0.01 as highly significant.

### 2.6. Manuscript Preparation

If not stated otherwise, calculations were performed in Microsoft Excel 2010 (Microsoft cooperation, Redmond, WA, USA), and final figures were prepared in Adobe Illustrator Version 25.2.3 (Adobe, San José, CA, USA).

## 3. Results

### 3.1. Efficacy of ABZ, Maca, and the ABZ+Maca Combination against AE in Mice

**Figure 3:**
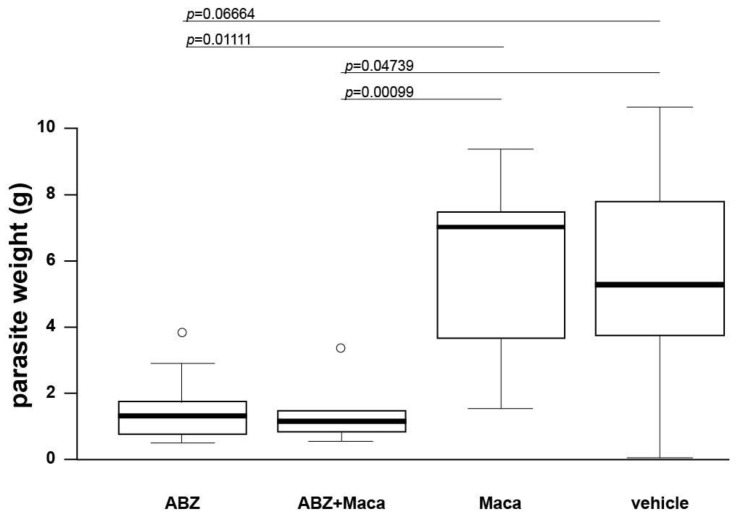
Effects of the different treatment regimens against secondary AE in mice. Intraperitoneally infected mice were treated with ABZ (200 mg/kg, 5 days per week, *n* = 9), ABZ+Maca (200 mg/kg ABZ and 33 mg/kg Maca, 5 days per week, *n* = 9), Maca alone (33 mg/kg Maca, 5 days per week, *n* = 9) or with the vehicle (corn oil, *n* = 8) for 6 weeks. At the end point of the study, parasite weight (g) was assessed. Wilcoxon rank sum test was applied for pairwise comparison of the treated groups, boxplots including outliers and *p*-values are given. Significant (*) and highly significant (**) changes are indicated.

The potential impact of the different treatments was assessed in a secondary AE mouse model by measuring the parasite weight recovered at the end of the treatment phase. As shown in Figure 3, ABZ treatment (*p* = 0.06664) and the combined treatment of ABZ+Maca (*p* = 0.04739) reduced the parasite weight as compared to the vehicle-treated group, whereas Maca alone did not affect the parasite mass (*p* = 1.0). One animal of the control group had to be euthanized early, as parasite tissue had grown around the intestine blocking the digestive tract. This datapoint was not included in the analysis.

### 3.2. Determination of Serum Levels of ABZ-Metabolites in the Different Treatment Groups

**Figure 4:**
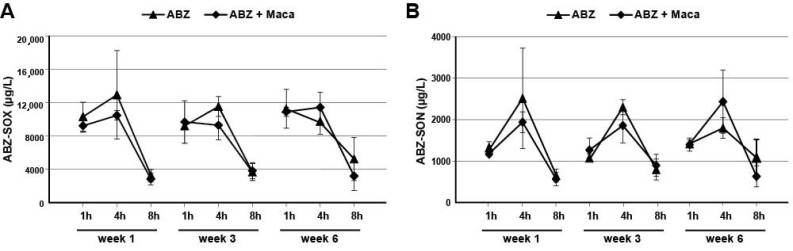
ABZ-metabolite levels in AE-mice. (**A**), ABZ-sulfoxide (ABZ-SOX) levels. (**B**), ABZ-sulfone (ABZ-SON) levels. Plasma levels were determined by UHPLC-MS/MS on treatment weeks 1, 3, and 6 in animals 1, 4, and 8 h after dosage. Plasma levels were determined in mice that were treated with ABZ alone, or in combination with Maca. Mean values and standard deviations are depicted for the three samples at each timepoint.

ABZ-sulfoxide (SOX) and ABZ-sulfone (ABZ-SON) levels were determined in *E. multilocularis*-infected mice treated with ABZ alone or ABZ in combination with Maca. As shown in Figure 4, the presence of Maca did not affect the ABZ-metabolite levels. ABZ-metabolite levels peaked at 4 h post dosing and dropped again after another 4 h. Over the course of the treatment of 6 weeks, ABZ-metabolites did not accumulate and reached similar levels over time.

### 3.3. Direct Impact of ABZ, Maca, and ABZ+Maca against E. multilocularis Metacestodes In Vitro

**Figure 5:**
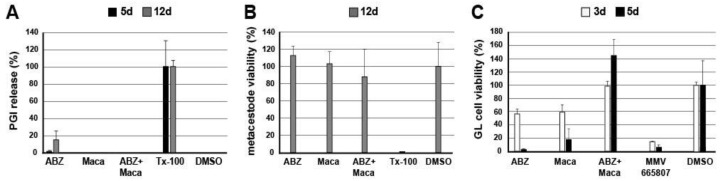
In vitro efficacy of ABZ and Maca against *E. multilocularis* metacestodes. The efficacy of ABZ (10.6 µg/mL), Maca (50 µg/mL) or a combination of both chemicals was assessed in vitro by (**A**) measurement of damage marker release by PGI assay; (**B**) metacestode viability assay by Alamar blue assay, and (**C**) GL cell viability testing by CellTiter-Glo assay. As a negative control, DMSO (0.1%) was applied, Tx-100 (0.1%) was applied as a positive control in (**A**,**B**), MMV665807 (0.3 µg/mL) in (**C**). Assays in (**A**,**B**) were performed in triplicate, in (**C**) in quadruplicate, and respective mean values and standard deviations are shown in relation to the negative control. Note the different incubation times applied for the different assays (**A**) 5 and 12 days; (**B**) 12 days; (**C**) 3 and 5 days.

To test whether Maca had any direct anti-metacestode activity, in vitro cultured *E. multilocularis* metacestodes were treated with ABZ, Maca or an ABZ+Maca combination. As shown in Figure 5A, ABZ treatment, particularly after 12 days of drug incubation, resulted in a slight release of PGI activity into the culture supernatant, which is indicative of physical metacestode damage. In contrast, Maca treatment did not induce any measurable release of PGI. In combination with ABZ, Maca reduced the activity of ABZ in vitro completely, as is visible after 12 days of treatment. To exclude any direct effects of Maca and/or ABZ on the enzymatic reaction applied for PGI detection, an additional test was performed (Figure S1). This test showed that neither Maca, ABZ, nor the combination of both, inhibited the enzymatic PGI reaction.

The viability of in vitro treated metacestode tissue, as assessed by Alamar blue assay, was not reduced by any of the treatments within 12 days of incubation when compared to the DMSO control (Figure 5B). However, the viability of extracted *E. multilocularis* GL cells was strongly reduced after 3 and 5 days of incubation with ABZ, as well as with Maca treatment alone. Interestingly, also here the ABZ+Maca combination treatment led to an increase in GL cell viability (Figure 5C). 

### 3.4. Ultrastructural Effects of ABZ, Maca and ABZ+Maca Treatments in E. multilocularis Metacestodes

**Figure 6:**
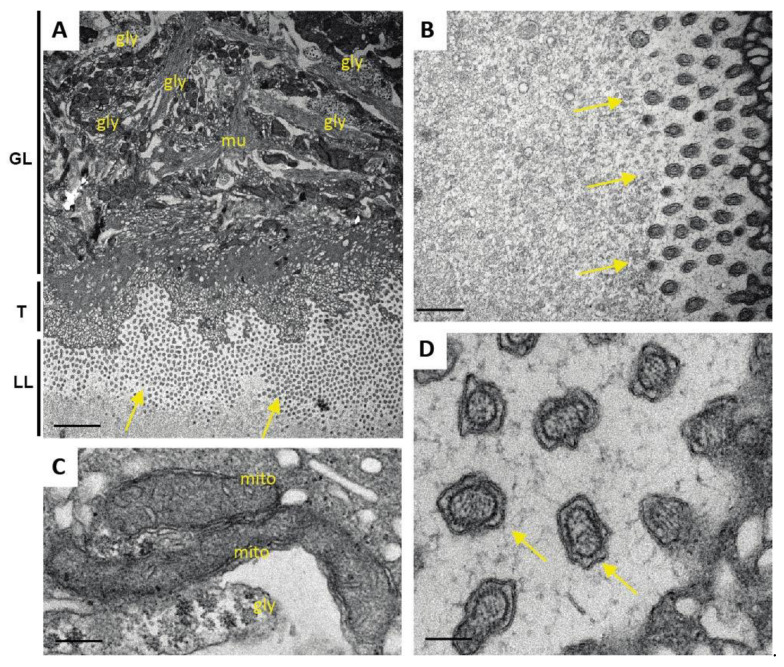
Structural features of *E. multilocularis* metacestodes. (**A**) low magnification view of a section through the metacestode wall, with laminated layer (LL), tegument (T), and germinal layer (GL) containing different cell types including glycogen storage cells (gly) and muscle cells (mu). Note that microtriches (arrows) are situated perpendicular to the section plane. (**B**) detailed view onto the T-LL interface with microtriches (indicated by arrows) and a more distal portion of the LL. Small vesicles are evident in the LL matrix. (**C**) High magnification view of two mitochondria (mito) exhibiting an electron dense inner matrix and cristae. Note the snowflake-like structures below the mitochondria, which indicate the presence of glycogen (gly). (**D**) High magnification view of cross-sectioned microtriches (arrows). The inner actin filament bundles form the microtriches cytoskeleton, which is surrounded by the outer membrane. Microtriches are embedded in a meshwork of thin filaments that form the matrix of the LL; mu = muscle cell. Bars in (**A**) = 3.5 µm; (**B**) = 0.7 µm; (**C**) = 0.25 µm; (**D**) = 0.15 µm.

Transmission electron microscopy (TEM) was used to evaluate the potential impact of ABZ, Maca, and ABZ+Maca treatments on metacestodes. When maintained in the absence of compounds, parasites exhibited typical features such as the outer, carbohydrate-rich laminated layer (LL), the tegument and the inner GL composed of multiple cell types including connective tissue, tegumental cytons, undifferentiated cells, glycogen storage cells, and muscle cells (Figure 6). Microtriches, which are formed by the tegument, protrude well into the LL and increase the resorbing, but also the secretory, surface of the parasite. Numerous exosome-like vesicles are released into the LL (Figure 6). 

**Figure 7:**
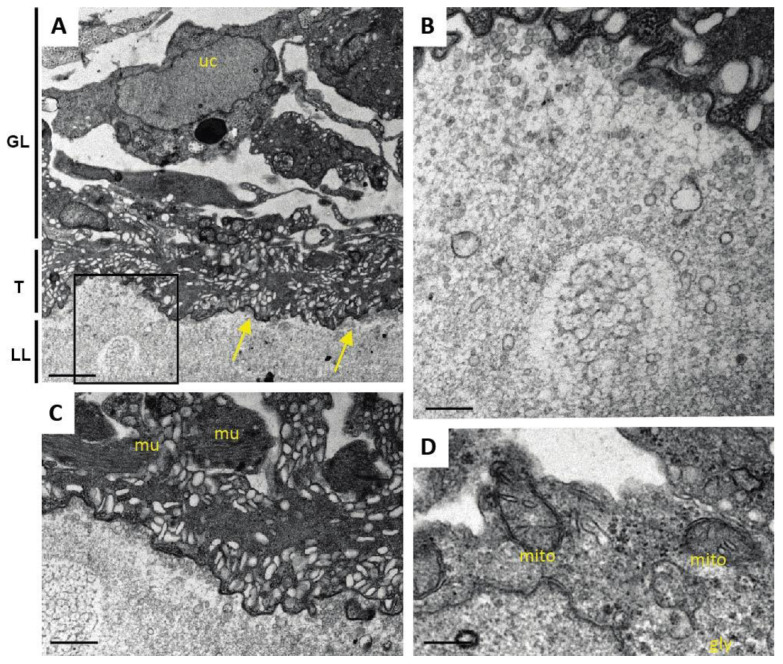
Ultrastructure of *E. multilocularis* metacestodes after 5 days exposure to ABZ in vitro. (**A**) Low magnification view showing the laminated layer (LL), tegument (T) and the germinal layer (GL) with an undifferentiated cell with a large nucleus (uc). Note the absence of microtriches at the LL-T interface (arrows). The boxed area in (**A**) is enlarged in (**B**). Note the small vesicles released by the tegument, which are continuously incorporated into the LL. (**C**) shows a higher magnification view of muscle cells (mu) and (**D**) shows structurally intact mitochondria (mito) embedded in the GL. Bars in (**A**) = 3.2 µm; (**B**) = 0.9 µm; (**C**) = 2.5 µm; (**D**) = 0.25 µm.

After 5 days of ABZ exposure (Figure 7), distinct alterations became evident especially at the interface between tegument and LL, with microtriches clearly shortened or even retracted. In contrast, the GL remained largely unaffected, and other structural features, such as the undifferentiated cells and muscle cells, also appeared intact. The fact that mitochondria were still unaltered under these conditions indicates that parasites were still viable (Figure 7).

**Figure 8:**
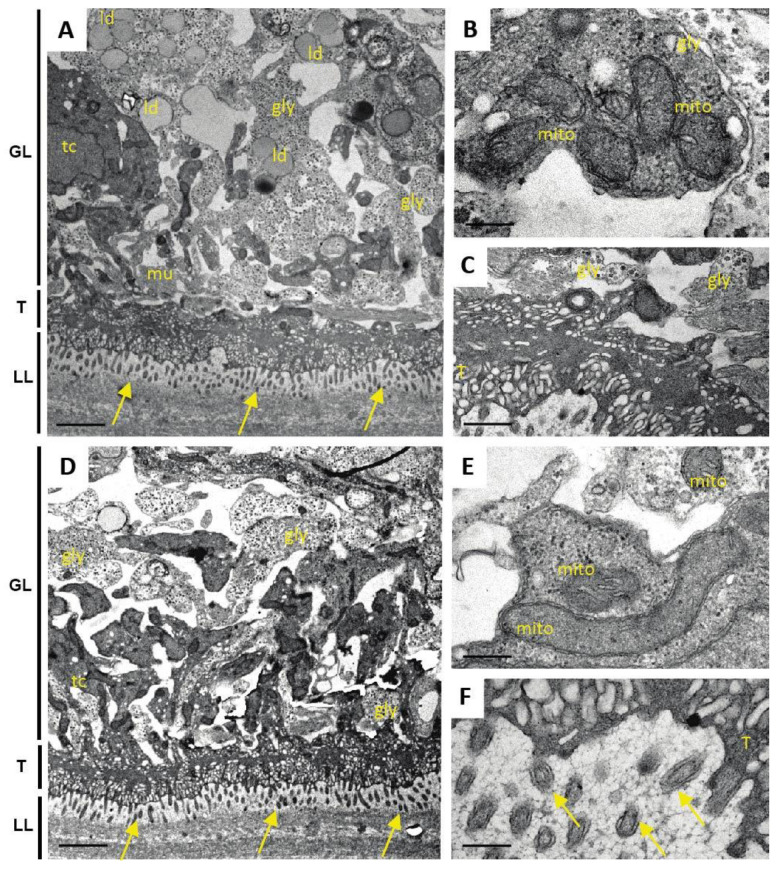
Ultrastructure of *E. multilocularis* metacestodes after 5 days exposure to Maca (A-C) and ABZ+Maca (D-F) in vitro. (**A**,**D**) Low magnification views showing the laminated layer (LL), tegument (T) and the germinal layer (GL). Microtriches are marked with arrows. Note that parasites are structurally intact; gly = glycogen storage cells; mu = mucle cells, ld = lipid droplets. (**B**,**C**,**E**,**F**) are higher magnification views of mitochondria (mito) (**B**,**E**) and the T-LL interface (**C**,**F**). Bars in (**A**,**D**) = 3.5 µm; (**B**,**E**) = 0.25 µm; (**C**) = 0.9 µm; (**F**) = 0.35 µm.

When studying the impact of Maca and ABZ+Maca treatments after 5 days (Figure 8), no structural differences could be noted to the control (Figure 6) with both treatments. Thus, Maca treatment did not induce any changes, and the treatment with ABZ+Maca did also not notably impact on the parasites, which exhibited structurally intact microtriches. Thus, the presence of Maca alleviated the effects of ABZ. 

**Figure 9:**
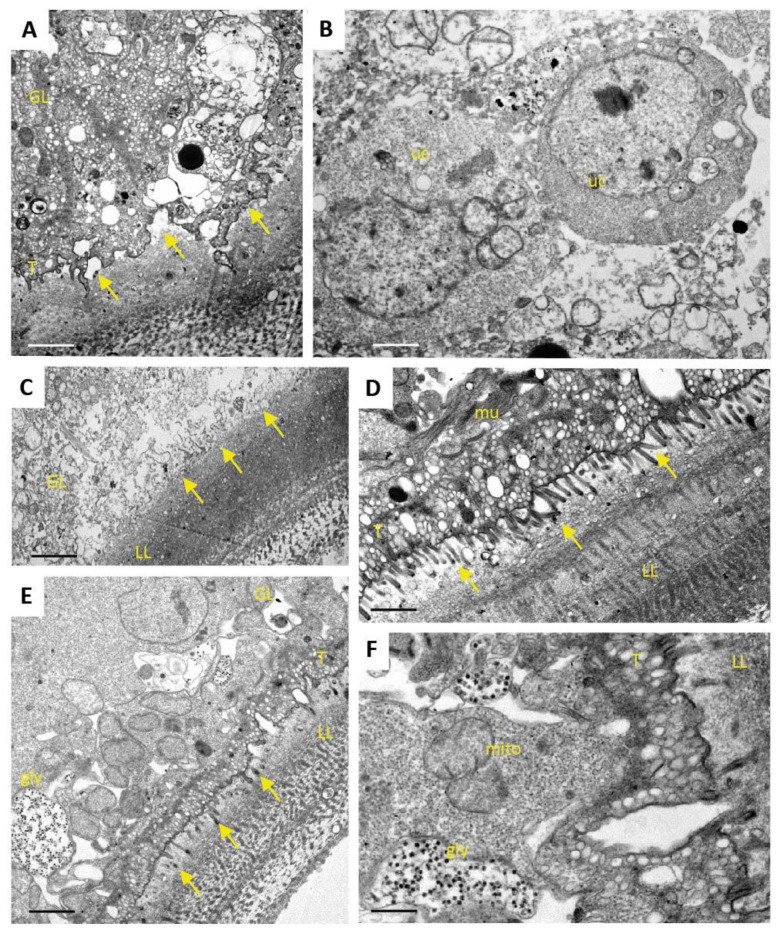
Ultrastructure of *E. multilocularis* metacestodes after 12 days drug exposure in vitro. Treatments were performed with ABZ (**A**–**C**), Maca (**D**) and ABZ+Maca (**E**,**F**) in vitro. ABZ treatment results in retraction of microtriches and partial detachment of tegument(T) and laminated layer (LL), and the germinal layer (GL) has a largely disorganized structure (**A**,**C**). However, undifferentiated cells are occasionally found that exhibit a largely intact morphology (**B**). Maca and Maca+ABZ treatment does not impact dramatically on the parasite ultrastructure, as seen with intact microtriches (arrows), glycogen storage cells (gly) and mitochondria (mito). Bars in (**A**) = 0.9 µm; (**B**) = 1.2 µm; (**C**) = 0.9 µm; (**D**) = 2.8 µm; (**E**) = 3.2 µm; (**F**) = 0.6 µm.

After 12 days of drug exposure (Figure 9), the effects of ABZ treatment were more pronounced and produced different results. The structural organization of the GL was clearly disturbed, and the GL appeared to partially separate from the LL, presumably due to the retraction of the microtriches. However, undifferentiated stem cells were visible, with seemingly still intact structural organization. In other parts of the metacestode, the tissue of the GL was largely destroyed and had lost its structural features, with only the LL being intact. In contrast, parasites treated with Maca or ABZ+Maca for 12 days appeared structurally unaltered, and even exhibited mitochondria with cristae-like features, indicating that these treatments had no, or only limited, impact on viability. 

### 3.5. Immunomodulatory Effect of Maca on Murine Spleen Cells

To check whether Maca could potentially exhibit direct immunomodulatory effects, we assessed the impact of treatment on naïve murine spleen cell viability by Alamar blue assay and on polyclonal activation and proliferation of Con A- and LPS-activated splenocytes by BrdU incorporation ELISA. 

**Figure 10:**
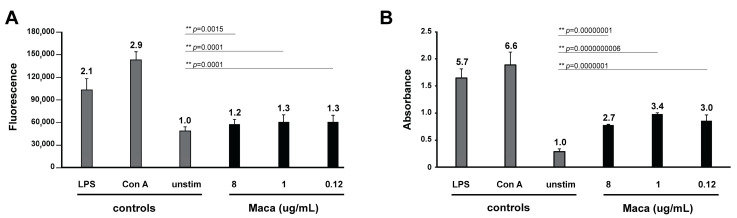
Immunostimulatory effects of Maca extract on murine splenocytes. (**A**) Alamar Blue assay for quantitative measurement of metabolic mitochondrial activity in splenocytes and (**B**) BrdU ELISA for quantitative assessment of BrdU incorporation by proliferating T- and B-lymphocytes. In (**A**,**B**), cultures of splenocytes were either left unstimulated, stimulated with Con A at 5 µg/mL, with LPS at 10 µg/mL or incubated with Maca extract applied at different concentrations (8, 1, and 0.12 µg/mL). Bars represent the mean signals of 5 replicates and standard deviations for Alamar and the mean absorbance values of 3 replicas and standard deviations for the BrdU ELISA. The numbers above the bars indicate the stimulation index (SI) in relation to the control unstimulated cells. *p*-values (Student’s t test) are given. Significant (*) and highly significant (**) changes are indicated.

As shown in Figure 10, Maca increased the metabolic mitochondrial activity of splenocytes dose-dependently and significantly by a factor of 1.2–1.3, this compared to unstimulated cells. Splenocytes stimulated with Con A or LPS were 2.1 to 2.9 times more metabolically active than unstimulated cells (Figure 10A). Furthermore, effects of Maca on B- and T-lymphocyte proliferation were directly assessed by measuring BrdU incorporation into DNA of dividing cells (Figure 10B). Control Con A- and LPS-stimulation resulted in multiplication of T- and B-cells by a factor of 6.6 and 5.7 times, respectively, compared to unstimulated splenocytes. Splenocytes incubated with Maca were also dividing, and dependent on the dose, they significantly incorporated 2.7 to 3.4 times more BrdU than unstimulated cells. 

Additionally, we assessed effects of Maca on Con A- and LPS-induced activation and proliferation of T- and B-lymphocytes (Figure S2). As demonstrated by Alamar Blue and BrdU ELISA, upon incubation with Maca, Con A- and LPS-induced T and B-cells maintained their fully enhanced metabolic mitochondrial activity and proliferative capability.

## 4. Discussion

Drug resistance is increasing in many parasites affecting humans and animals, and vaccines are rare. For some parasites such as *E. multilocularis*, no curative drugs are available. In alternative approaches, medicinal plants and in particular some of their secondary metabolites have been appraised as a possible source for anti-parasitic agents [14,26]. Many studies have reported on the in vitro effects of plant extracts against protozoan and helminth parasites. However, rarely have they been followed up in animal models, or even in clinical trials.

To date, only few studies applied plant extracts or products against *E. multilocularis*: thymol, as major component of the essential oils from *Thymus vulgaris* and *Origanum vulgare,* and anacardic acid from Brazilian cashew-nut shell liquid were successfully tested against AE in mice [27,28]. Ampelopsin extracted from moyeam, anacardic acid from Brazilian cashew-nut shell liquid, and *Thymus capitatus* essential oil exhibited in vitro activity against *E. multilocularis* metacestodes [28,29,30]. A larger number of compounds have been assessed for activity against the closely related parasite *E. granulosus* [31]. A major drawback in most of these studies is that toxicological profiles have never been assessed, and promising candidates are not further followed to reach clinical application in AE patients. 

The Peruvian root Maca has been cultivated and consumed (as food) by indigenous Andeans for millennia. Apart from its caramel-like aroma, it is appraised for comprising several essential nutrients (amino acids, vitamin C, copper, iron, etc.), and also bioactive compounds, which could be of human health benefit [32]. For this reason, Maca has received increasing marketing promotion and attention over the last two decades as a dietary supplement. It is sold in different forms, from powder to pills and liquors [32]. Overall, Maca has been shown to be able to help stimulate sexual dysfunction, it has neuro- and cancer-protective effects, and acts as an anti-depressant, anti-oxidant, and anti-inflammatory agent. Secondary metabolites contained in Maca are alkaloids, glycosides, tannins, saponins, polyphenols, macamides, macaenes, and macahidantoins [33].

We here report on a patient suffering from AE who was treated with ABZ, and was unexpectedly no longer showing signs of vital AE after 3 years of therapy. It was found that during ABZ therapy this patient had been taking Maca. AE is a severe and chronic disease, and curative treatment can usually only be achieved by radical surgery. Liver transplantation is only considered as the ultimate therapeutic procedure. The patient described in this report could not undergo radical surgery, but received biliary plastic stents, which were replaced on a regular basis for 2 years. Otherwise, the patient received standard ABZ therapy. After 42 months, a complete disappearance of viable *E. multilocularis* microcysts was observed, followed by Em18 antibody negativation and perilesional activity extinction on PET-CT. All these results indicate that metacestode viability was severely impaired. After an additional 3 years of ABZ (and Maca) therapy, it was decided to interrupt the treatments under close surveillance. As the patient self-treated himself with a constant and daily dose of Maca, we decided to follow up on the potential anti-parasitic properties of this root.

The usual procedure for the identification of novel drugs against infectious diseases is a forward approach. This includes extensive in vitro activity testing, in vitro host toxicity assessment, and assessment of efficacy in a mouse model and/or possibly a large animal model. Finally, in case of promising outcomes, application as salvage treatment in human patients would be considered [12]. However, here we present a reverse approach, which started with a positive disease development in a human patient undergoing ABZ+Maca therapy, then testing in an in vivo mouse model, and further characterization of potential anti-parasitic activity of Maca or ABZ+Maca treatments in vitro.

In the in vivo AE mouse model, which is a secondary infection model that is often applied for first-line testing of putative anti-AE compounds [12], Maca did not exhibit any anti-parasitic effects, whereas ABZ treatment led to the expected outcome. Future studies could include testing of Maca in a primary egg-infection infection model. However, such eggs are rarely available for research projects, and they also pose a high infectious risk for the experimenters.

As an additional parameter, effects of Maca on ABZ metabolite-levels were investigated, as past studies showed that combining ABZ with other drugs might influence the serum levels and the half-life of ABZ metabolites [34]. A novel UHPLC-MS/MS based method was established to measure ABZ-SOX and ABZ-SON levels in the small sample volumes generated from mouse studies. However, no effects of Maca on ABZ metabolite plasma levels were observed. In any case, this UHPLC-MS/MS assay will allow to assess effects of any treatment on ABZ metabolite-levels in future studies, and is therefore an important addition to the repertoire of test methods available for the investigation of potential anti-echinococcal compounds.

As many factors influence the activity of a compound in vivo, we went one step back in the screening cascade and applied several well-established in vitro tests to investigate the effects of Maca against *E. multilocularis*. Overall, none of these tests provided any evidence for anti-parasitic properties of Maca. In contrast, ABZ again led to the expected drug-induced changes. It is of note here that a relatively long drug incubation period had to be chosen, as the standard drug in use, ABZ, is effective only at a slow pace when applied in vitro. Additionally, Maca was also tested for effects on isolated *E. multilocularis* GL cells, and here not only ABZ, but also Maca, were active. Thus Maca is, possibly non-specifically, cytotoxic against isolated GL cells, but not against GL cells that are protected by a surrounding LL, as is the case in an infected AE patient. Strikingly, in all the in vitro assays we could show that Maca interfered in the treatment effect of ABZ. Potentially, a drug–drug interaction could lead to loss of activity in the in vitro setups, but a similar effect was not observed in the mouse model. However, caution should be taken for human application of Maca, as a respective drug–drug interaction in human AE patients cannot be ruled out. The topic of drug–drug interaction was not further investigated within the scope of the present study.

Summarizing, none of our experiments could prove any direct anti-parasitic activity of Maca. There are no reports of previous studies on Maca against *Echinococcus* [35]. One study had investigated the effects of extracts of the related *Lepidium sativum* against *E. granulosus* and found in vitro scolicidal activities, but the applied concentrations were rather high (10 mg/mL) and therefore possibly unspecific. 

However, besides direct anti-parasitic activity, Maca could induce indirect effects that cannot be picked up in vitro. There are several studies showing that Maca has effects on the immune system. In the leukemic RAW264.7 cells, Maca was shown to promote proliferation, phagocytosis, secretion of nitric oxide, TNF-alpha, and IL-6, and enhanced expression of CD80 [36,37]. Maca was also shown to remodel the immunosuppressive tumor microenvironment into an immune-activated state with secretion of IL-12, TNF-alpha, and IFN-gamma [38]. Further, Maca can promote proliferation of CD4+ T cells [39]. For these reasons, Maca was proposed as a supplement to activate the immune response, and for cancer immunotherapy [37,38,40]. Our data also confirmed the immunostimulatory ability of Maca, and we could at the same time not detect any immune-inhibitory effects of Maca on T- or B-cells.

Given the resemblances of the *E. multilocularis* metacestode building up an immunomodulatory surrounding in its host, immunotherapy has been investigated for future treatments of AE patients [41]. Whether Maca indeed would be able to induce such an immunological change leading to clearance of AE infection in an infected patient was not assessed in this study.

## 5. Conclusions

Neither our studies in an AE mouse model nor the in vitro assays showed an anti-parasitic effect of Maca against *E. multilocularis*, with the exception of cytotoxic activity of Maca against isolated GL cells. The good treatment response described for one patient self-medicating with Maca in addition to ABZ thus cannot be explained by a direct anti-parasitic activity in the plant extract. It remains, however, to be elucidated whether the plant extract induced immunomodulatory effects leading to the good treatment response.

## Figures and Tables

**Table 1:** Summary of MS/MS parameters for the analytes and their corresponding internal standards.

Name	Parent Ion [m/z]	Product Ion [m/z]	Cone [V]	Collision [V]
ABZ-SOX quantifier	282.1	208.2	40	23
ABZ-SOX qualifier	282.1	240.2	40	12
ABZ-SON quantifier	298.1	159.2	60	35
ABZ-SON qualifier	298.1	224.2	60	25
[^2^H_7_]-ABZ-SOX quantifier	289.1	209.2	40	23
[^2^H_7_]-ABZ-SOX qualifier	289.1	241.2	40	12
[^13^C,2H_3_]-ABZ-SON quantifier	302.1	159.2	60	35
[^13^C,2H_3_]-ABZ-SON qualifier	302.1	224.2	60	25

## Data Availability

Not applicable.

## Supplementary Materials

The following are available online at https://www.mdpi.com/article/10.3390/pathogens10101335/s1: Figure S1. PGI inhibition test; Figure S2. Immunomodulatory effects of Maca extract on Con A- and LPS-induced splenocytes.
