# Supplementary figures and images for: Maca against Echinococcosis?—A Reverse Approach from Patient to In Vitro Testing
Source: Pathogens. 2021 Oct 15;10(10):1335. doi: 10.3390/pathogens10101335 (PMC8537204; doi:10.3390/pathogens10101335)

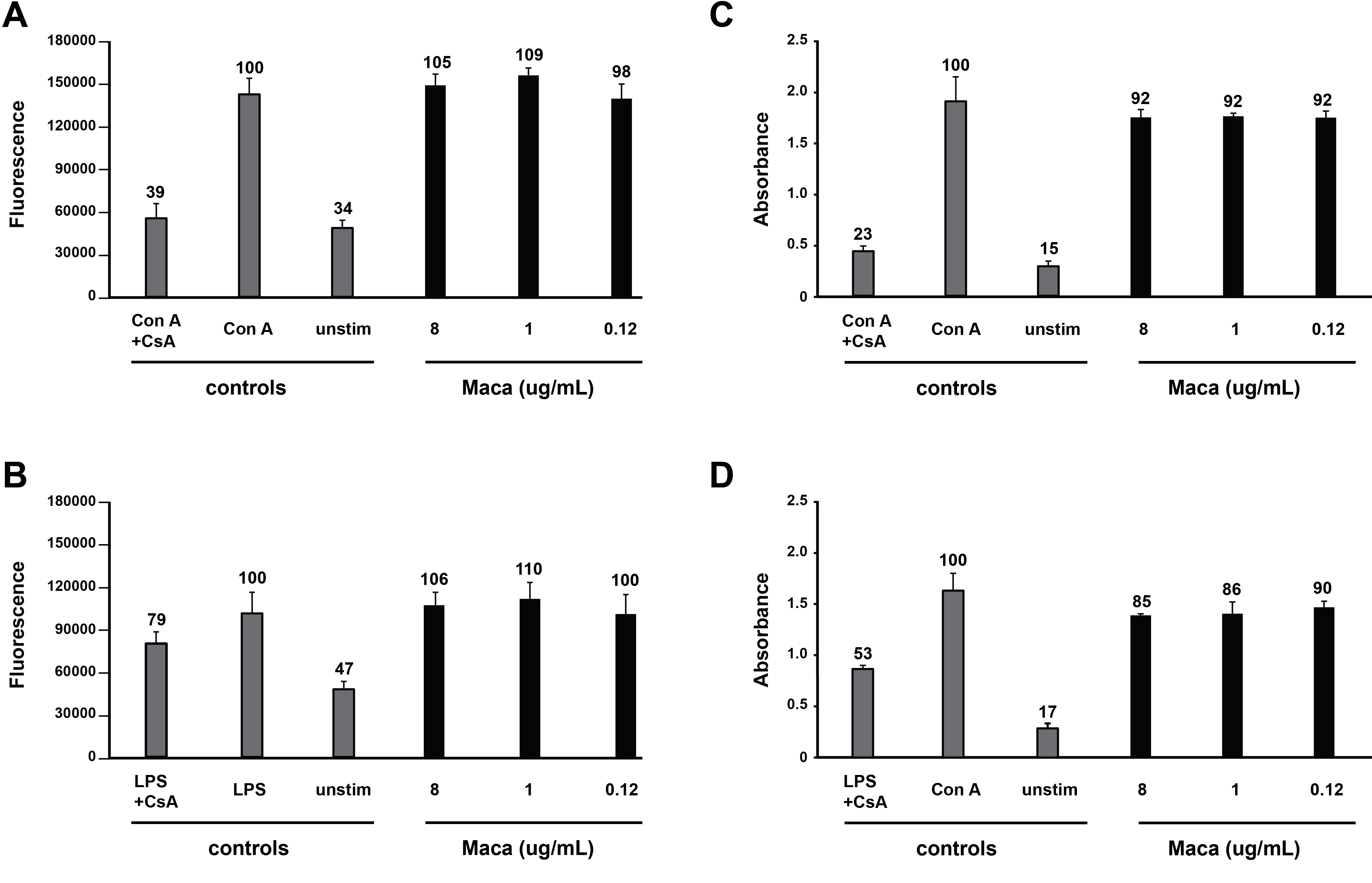

Supplement: Supplementary file 1 [file pathogens-10-01335-s001.zip › Figure S2.jpg]

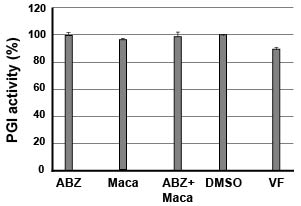

Supplement: Supplementary file 1 [file pathogens-10-01335-s001.zip › Figure S1.jpg]
